# A Retrospective Study of Two-port Appendectomy and its Comparison with Open Appendectomy and Three-port Appendectomy

**DOI:** 10.4103/1319-3767.70611

**Published:** 2010-10

**Authors:** Vipul D. Yagnik, Jignesh B. Rathod, Ajay G. Phatak

**Affiliations:** Department of Surgery, Pramukh Swami Medical College, Shree Krishna Hospital, Karamsad - 388325, Gujarat, India

**Keywords:** Laparoscopy, appendectomy, two-port technique

## Abstract

**Background/Aims:**

To establish the efficacy of two-port appendectomy as an alternative to standard laparoscopic and open appendectomy in the management of acute appendicitis.

**Materials and Methods:**

Of the 151 patients included in the study, 47 patients were in the open group, 61 in two-port and 43 patients were included in the three-port group. Only patients with uncomplicated acute appendicitis were included in the study. Patients with complicated appendicitis like perforated appendix, appendicular lump and appendicular abscess were excluded from the study. Patients converted to open procedure after initial diagnosis and patients with other pathology in addition to appendicitis were also excluded. Patients with recurrent appendicitis and chronic appendicitis were excluded. The total number of excluded cases was 50. Data were compared with cases of open and three-port appendectomy.

**Results:**

The mean operative time was 43.94, 35.74, and 59.65 min (SD: 18.91, 11.06, 19.29) for open, two-port, and three-port appendectomy groups respectively. Mean length of stay in days was 3.02, 1.93, and 2.26 (SD: 1.27, 1.04,1.09) for open, two-port, and three-port appendectomy groups respectively. Surgical site infection was significantly lower (*P* = 0.03) in laparoscopy group as compared to that in open appendectomy group. Seven patients (4.63%) developed surgical site infection, 5 (10.63%) in the open and 2 (1.92%) in the laparoscopy group. Surgical site infection was 1.63% and 2.32% in two-port and three-port appendectomy groups respectively.

**Conclusions:**

For uncomplicated appendicitis, the two-port appendectomy technique significantly reduces operative time as well as length of hospital stay. It also reduces surgical site infection as compared to open appendectomy group.

The first laparoscopic appendectomy was performed by Semm in 1982.[1] But laparoscopic appendectomy is still to stand its ground against open appendectomy, because of overall cost involved in the treatment as well as higher rate of intraabdominal infection.[2] However, the advantages of laparoscopic appendectomy are lesser postoperative pain, lesser incidence of surgical site infection and shorter hospital stay. Recent EAES (European Association of Endoscopic Surgery) guidelines state that laparoscopic appendectomy has a small but definite advantage over open appendectomy.[3] Recent innovations in technique like extracorporeal appendectomy have been associated with shorter operative time and short learning curve.[4] Further, an unstated advantage of laparoscopic technique is inspection of whole peritoneal cavity with excellent clarity. The aim of this study is to compare the various techniques of appendectomy like open, two-port and three-port as well as their outcome in terms of operative time, postoperative pain, length of hospital stay and surgical site infection.

## MATERIALS AND METHODS

A retrospective study was carried out involving patients operated at the Shree Krishna Hospital, Pramukhswami Medical College, Karamsad, during the period March 2007–March 2009. The analysed group consisted of patients with uncomplicated acute appendicitis. Patients with complicated appendicitis like perforated appendicitis, appendicular abscess, lump and those with other intraabdominal pathology, in addition to appendicitis, were excluded from the analysed group. Patients converted to open procedure after initial diagnosis were also excluded from the study. All patients underwent laparoscopic appendectomy under general anesthesia by surgeons qualified in doing laparoscopic appendectomy. Clinical assessment was confirmed by complete blood counts, and ultrasonography report. Outcome was assessed in the form of operative time, length of hospital stay and postoperative complications. Preoperatively, all patients were well hydrated with balanced salt solution. Antibiotics were administrated preoperatively to cover gram-negative and anaerobic organisms.

### Technique

**Figure 1:**
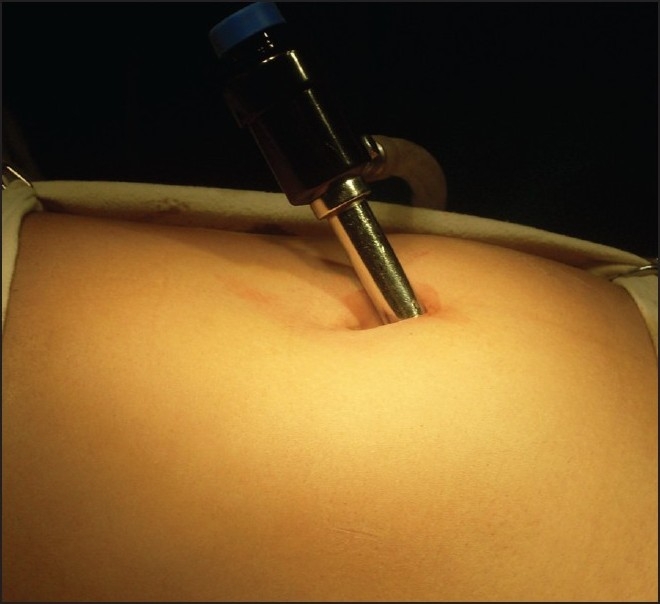
First sub umbilical trocar for accommodation of camera

**Figure 2:**
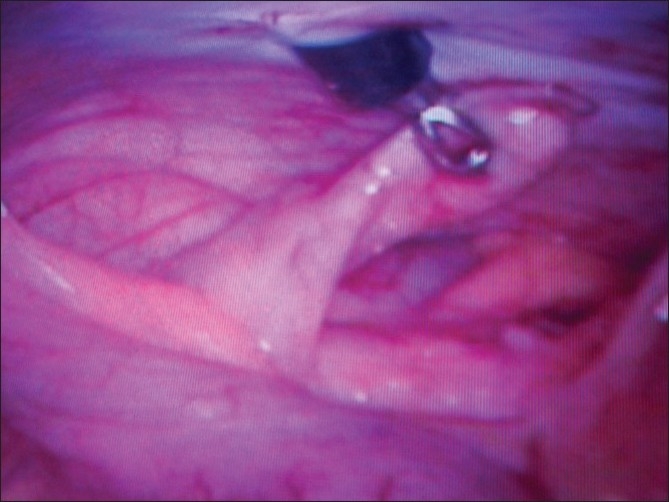
Appendix grasped with Babcock forcep

**Figure 3:**
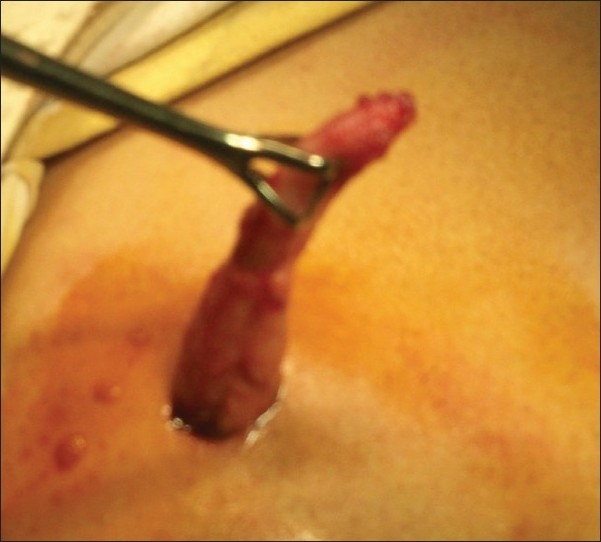
Appendix delivered through 10 mm right lower quadrant incision

After decompression of bladder with per urethral catheterization, pneumoperitoneum was created in a standard manner with Veress needle in infraumbilical position. A 10 mm trocar was inserted for accommodating telescope [Figure 1] and another 10 mm port was inserted by looking at the position of the appendix in the right lower quadrant, or if the appendix was not seen easily then the port was inserted at the McBurney’s point. Appendix was identified by using the standard technique. It was grasped with either the Babcock forceps or a bowel grasper [Figure 2]. Appendix was delivered through the right lower quadrant port [Figure 3]; pneumoperitoneum was deflated and appendicular artery was ligated on the free border of mesentery with Vicryl 2-0 and cut. Appendix was ligated with Vicryl 2-0, approximately 1.25 cm distal to the base. Another ligature was applied distal to first ligature with adequate space in between to cut the appendix. Hemostasis was checked with scope at the end. Closure of the umbilical as well as right iliac fossa port was done by Vicryl 2-0 and skin was closed with skin stapler. All appendectomy specimens were sent for histopathological examination. Patients for the open, two-port or three-port group were selected randomly from the uncomplicated appendicitis. All patients were followed up 1 month to look for surgical site infections.

## RESULTS

A total of 201 patients were operated during March 2007–March 2009 in Shri Krishna Hospital, Karamsad. Out of these, 50 patients were excluded, because of not fulfilling the criteria laid down for inclusion in the study. Results obtained from the study are shown in Tables 1–4.

**Table 1:** Demographic characteristics of the patients undergoing appendectomy

Parameter/technique used	Open (*N* = 47)	Two-port (*N* = 61)	Three-port (*N* = 43)	Total (*N* = 151)	*P* value
Mean age	23.34	22.67	24.53	23.41	0.69*
(SD)	(13.44)	(9.65)	(9.61)	(10.91)	
Female patients	15	22	12	49	0.68**
(% of total)	(31.9)	(36.1)	(27.9)	(32.5)	

* *P* value was calculated using ANOVA;

** *P* value was calculated using Chi-squared test

**Table 2:** Comparison of operative time of various appendectomy techniques

Technique used	*N*	Mean	SD	*P* value
Open	47	43.94	18.91	< 0.0001
Two-port	61	35.74	11.06	
Three-port	43	59.65	19.29	
Total	151	45.1	18.96	

Mean and SD values are in minutes

**Table 3:** Length of hospital stay for patients operated using different surgical techniques

Technique used	*N*	Mean	SD	*P* value
Open	47	3.02	1.27	0.00001
Two-port	61	1.93	1.04	
Three-port	43	2.26	1.09	
Total	151	2.36	1.22	

Mean and SD values are in minutes

**Table 4:** Comparison of post-op infection in patients operated using open and laparoscopic appendectomy techniques

Technique used	Post-op. infection (Yes/NO)		Total
	Number	Yes	No.
Open	42	5	47
Two-port and three-port combined	102	2	104
Total	144	7	151

*P* = 0.03

As the *P* value is greater than 0.05 for the parameters age and gender, it suggests that there is no baseline difference in relation to parameters age and sex. So the result we have obtained is probably because of the effect of the treatment. The limitation of the study is that we have recorded only age and sex for comparison; there may be other important variables like body weight and nutritional status of the patients, that were not taken into consideration, but could have made a difference.

Statistically significant differences were noted in the operative time. The operative time is significantly lower (*P*=0.03) in the two-port technique, while it is significantly higher (*P* = 0.001) in the three-port technique as compared to open technique using Dunnet’ post-hoc comparison.

There was a highly statistically significant difference in the hospital length of stay among the three groups (based on ANOVA). The post-hoc comparison using Dunnet’s t-test revealed that patients operated using two-port (*P* < 0.001) and three-port (*P* = 0.009) techniques had significantly lesser hospital stay as compared to that for patients operated using open technique.

As the infection rate is very low, we used Fisher exact test to obtain the *P* value. There was a statistically significant difference in the surgical site infection. The infection was significantly lower (*P* = 0.03) in laparoscopic group in comparison to open appendectomy.

## DISCUSSION

New concepts in surgery are not always accepted readily; these usually meet with an initial resistance. A great example of this has been laparoscopic surgery. The role of laparoscopic appendectomy as compared to open appendectomy is still controversial. But the former has proved to be clearly beneficial in obese as well as women of reproductive age group, and in patients with diagnostic dilemma. The laparoscopic appendectomy is also gaining popularity because of shorter operative time, lesser postoperative pain and lesser incidence of surgical site infection. Laparoscopic appendectomy has now become an indispensible tool for treatment of those with undiagnosed abdominal pain for diagnostic workup. Laparoscopic appendectomy is now considered to be a safe and excellent alternative to open appendectomy. Its only drawback is a slightly higher rate of intraabdominal abscess.[5–8] Complicated appendicitis and poor risk for general anesthesia are considered to be relative contraindications for laparoscopic appendectomy. Nowadays, attempts have been made to reduce the number of ports and improve cosmesis.[9] The two-port technique is similar to the three-port technique, except that appendix is delivered through right iliac fossa 10 mm port and tied extracorporeally and removed. This technique was utilized by us for patients of all age groups. A few randomized controlled trials show that laparoscopic appendectomy is safe and effective for treatment of appendicitis with improvement in outcome.[10] Two-port technique has an added advantage of minimal tissue trauma. Traditional laparoscopic appendectomy (three-ports) did not offer much advantage over the open appendectomy due to prolonged operative time and higher cost.[2] Open appendectomy still confers benefit in terms of lesser incidence of intraabdominal abscess.[5,6,7,8] Use of laparoscopy or laparoscopic appendectomy is generally recommended in patients with suspected appendicitis unless laparoscopy itself is a contraindicated or not feasible.[11] According to Cochrane review published in 2004, there is certain advantage of laparoscopic appendectomy over open appendectomy, but the advantage is not large enough.[11] Routinely, laparoscopic appendectomy is performed using three-ports. Some surgeons have recommended use of single-port or two-port technique. In our study, we have used two-port techniques as mentioned above. In this study, mean age of the patients was 22.67 years for two-port group. The mean operative time was 35.74 min, which is comparable to that reported in a study done by Gohary *et al*. (34.4 min).[12] Adhikary *et al*. have reported a mean operative of 23.3 min.[13] The average length of hospital stay was 1.93 days in our study. It was less compared to that reported in studies done by Adhikary *et al*.[13] (2.4 days) and Gohary *et al*.[12] (3.4 days). Early discharge in our study was probably because patients had lesser pain. One patient developed surgical site infection (2%) in our study with no intra-abdominal abscess. Gohary *et al*.[12] reported 0%, while Adhikary *et al*.[13] reported 10% surgical site infection.

We found statistically significant difference between outcomes of various techniques of appendectomy. Operative time was significantly lower (*P* = 0.03) in two-port and significantly higher (*P* = 0.001) in three-port technique as compared to open appendectomy. Short-operative time in two-port technique was probably because of ease of operative technique and extracorporeal knotting being easier and faster. There are two basic technique for three-port appendectomy 1) Endo loop – preformed knot technique and 2) Endo GIA stapler. In rural set-up, where we are practising, it is not feasible to use these techniques because of cost constraint. We found statistically significant difference in the length of hospital stay in laparoscopic group (*P* < 0.001 for two-port and *P* = 0.009 for three-port) as compared to open appendectomy technique. Patients in the laparoscopy as well as open group belong to uncomplicated appendicitis. Early discharge from the hospital was probably because of lesser postoperative pain and early return of bowel movement.

In our study we found statistically significant difference in surgical site infections. Surgical site infection was significantly lower (*P* = 0.03) in laparoscopy group as compared to that in open appendectomy group. Seven patients had developed surgical site infection. Out of these seven patients, five belonged to open and two to laparoscopy group. Surgical site infection was 1.63% and 2.32% in two- port and three-port appendectomy groups’ respectively. There was no case of intraabdominal abscess in any group, probably because we included only uncomplicated acute appendicitis in this study. Though appendix is in the trocar hole and is inflamed, surgical site infection is not higher probably because of preoperative prophylactic antibiotics we had used. For patients with uncomplicated appendicitis, a single preoperative dose of antibiotic was given, that covered both the aerobic and anaerobic colonic flora reduces surgical site infection and intraabdominal abscess formation[14]

Laparoscopic appendectomy, especially two-port, is found to be cost effective because of shorter operative time, significant early discharge from the hospital and lesser surgical site infection. Psychological trauma associated with prolonged dressing and cost involved with routine dressing can also be avoided.

## CONCLUSION

For uncomplicated appendicitis the two-port appendectomy technique has been found to be very useful in retrospective comparative analysis with three-port and open appendectomy techniques. Two-port appendectomy has been found to be associated with significantly shorter operative time, lesser incidence of surgical sites infection, lesser postoperative pain and significantly lesser hospital stay.
